# Morphology, Morphometry, and Immunohistochemical Profile of Megakaryocytes and Bone Marrow Microenvironment in Disease Progression and Therapy Resistance in Chronic Myeloid Leukemia

**DOI:** 10.7759/cureus.67772

**Published:** 2024-08-25

**Authors:** Sreerag Kana, Debdatta Basu, Rakhee Kar, Rajesh Nachiappa Ganesh, Biswajit Dubashi, Harichandrakumar KT

**Affiliations:** 1 Pathology, Jawaharlal Institute of Postgraduate Medical Education and Research, Puducherry, IND; 2 Pathology, Mahatma Gandhi Medical College and Research Institute, Puducherry, IND; 3 Medical Oncology, Jawaharlal Institute of Postgraduate Medical Education and Research, Puducherry, IND; 4 Biostatistics, Jawaharlal Institute of Postgraduate Medical Education and Research, Puducherry, IND

**Keywords:** cd44, morphometry, megakaryocyte, chronic myeloid leukemia, microenvironment, bone marrow

## Abstract

Background

Tyrosine kinase inhibitors have revolutionized the treatment of chronic myeloid leukemia (CML) since the beginning of the century. However, resistance to therapy and the progression of disease tend to occur in certain patients. The bone marrow microenvironment may play a role in the disease outcome. Megakaryocytes have multiple roles in the regulation and maintenance of the hematopoietic stem cell microenvironment. In the current study, we evaluated the association of megakaryocyte morphology, morphometry, and microenvironment with disease progression and therapy resistance in CML.

Methodology

Megakaryocyte morphology and morphometry were analyzed and compared between the different phases (chronic and advanced) at diagnosis in 150 cases of BCR-ABL-positive CML. All CML-CP patients (n = 119) were followed up on tyrosine kinase inhibitor therapy for a minimum of 15 months and classified based on their treatment outcome as a response, resistance to therapy, or progression of disease based on standard criteria. Immunohistochemistry on a bone marrow trephine biopsy was done for vascular endothelial growth factor (VEGF), FOXP3, CD150, CD48, CD44, osteopontin, CXCL12, N-cadherin, PDL-1, and IL-7, and their expression on megakaryocytes and their association with treatment outcome was evaluated.

Results

The morphology and morphometry of megakaryocytes showed a heterogeneous population in CML. Morphology and morphometric parameters, when compared between the chronic and advanced phases of disease at diagnosis, did not show any statistical difference. Megakaryocytes were variably positive for VEGF, FOXP3, CD150, CD48, osteopontin, N-cadherin, CXCL12, CD44, PDL-1, and IL-7. However, only CD44-positive megakaryocytes were statistically associated with the treatment outcome. The patients with a higher expression of CD44 megakaryocytes progressed to the advanced phase of the disease during therapy compared to those who responded.

Conclusion

Megakaryocyte morphology and morphometry were heterogeneous in CML; however, they did not show any significant difference with either the phase of the disease or with treatment outcomes. Among the various immunohistochemical markers of the microenvironment, only CD44-positivity on megakaryocytes was associated with poor treatment outcomes.

## Introduction

Chronic myeloid leukemia (CML) is a common malignancy of the blood and bone marrow [1]. At the beginning of this century, tyrosine kinase inhibitors (TKI) were developed, and Imatinib was the first TKI approved for therapy [2]. While the majority of patients diagnosed with CML responded well to Imatinib, resistance developed in a few, leading to treatment failure and relapse [3]. Resistance to treatment is multifactorial, with either BCR-ABL-dependent or independent mechanisms [4].

The bone marrow microenvironment (BMM) is regarded as a safe haven for hematopoietic stem cells (HSCs) and is composed of various niches represented by molecules and cytokines. HSCs proliferate and develop into all the lineages, whether myeloid or lymphoid, and are responsible for creating the entire hematopoietic and immune systems [5,6]. The megakaryocyte in the bone marrow matrix has various roles, ranging from the production of platelets to supporting and regulating the hematopoietic niches by secreting growth factors [7-10].

In this study, we aimed to evaluate the megakaryocyte morphology, morphometry, and expression of markers representing BMMs such as CD44, CD48, CD150, vascular endothelial growth factor (VEGF), FOXP3, N-cadherin, osteopontin, PDL-1, and IL-7 and their association between disease progression and therapy resistance in CML.

## Materials and methods

The present study was a nested case-control study carried out at Jawaharlal Institute of Postgraduate Medical Education and Research, Puducherry in southern India. Institutional Ethics Committee for Observational Studies, Jawaharlal Institute of Postgraduate Medical Education and Research, Puducherry issued approval JIP/IEC/2019/0187.

Newly diagnosed patients with CML irrespective of the phases, were included and evaluated prospectively. All BCR-ABL-negative CML cases and patients who were already on therapy were excluded from the study. The cases were categorized as CML in the chronic phase (CML-CP) and CML in the advanced phase (CML-AdP) [11].

Bone marrow was collected as a part of the diagnostic workup and was used for the study after obtaining informed consent from the study participants.

A bone marrow biopsy was fixed and decalcified based on the Hammersmith protocol [12]. Three µm sections were made from the paraffin-embedded sections. The sections were then stained with hematoxylin and eosin (H&E), Masson’s trichrome, and Gomori’s stain for collagen and reticulin, respectively, and immunohistochemical stains by using standardized laboratory protocols.

The morphology and morphometry of megakaryocytes were analyzed on H&E-stained tissue sections. Morphologically, megakaryocytes were counted per high power field, and their detailed appearance, whether normal, dwarf, or enlarged, was noted. The nuclear lobations were also noted. Morphometry was done by using an Olympus microscope and the image analysis software ProRes CapturePro v2.8.8. The following parameters were studied: Megakaryocyte count, which was estimated per high power (400x) field and then converted into numbers per square millimeter area, cytoplasmic major diameter, cytoplasmic area, nuclear major diameter, and nuclear area. The nuclear-to-cytoplasmic ratio (N:C ratio) was calculated from the nuclear area and cytoplasmic area.

Bone marrow immunohistochemistry

Immunohistochemistry was performed on 2-3 µm acetic zinc formalin (AZF)-fixed paraffin-embedded sections made on the positively charged slides.

The research-use-only markers like CD25, FOXP3, osteopontin, CXCL12/SDF-1, CD44, CD150, N-cadherin, and IL-7, which were standardized for the antigen retrieval method and medium, as well as the pH of the retrieval medium. For staining markers such as CD34, CD61, CD41, CD4, and CD48, an available standardized departmental protocol was used. Antibody titration was done for all the concentrated antibody markers. All concentrated antibodies were diluted in a 1% bovine serum albumin solution. Only CD34, VEGF, and CD61 were ready-to-use, prediluted antibodies.

PDL-1 staining was done on Roche Ventana Bench BenchMark GX (Roche, Basel, Switzerland) using PDL-1 clone SP-263 and the secondary kit, the Optiview DAB Detection Kit (Roche, Basel, Switzerland).

The pattern of staining was noted in all the cases, and even one megakaryocyte positive for the marker was considered positive. Appropriate negative and positive controls were used to compare the positivity.

Follow-up

The patients in CML-CP were followed up for a minimum of 15 months to monitor treatment outcomes during the therapy. The treatment monitoring was done by quantitative real-time PCR for measuring BCR-ABL1 mRNA in peripheral blood during Imatinib therapy. The response was assessed using European LeukemiaNet (ELN) criteria [13]. Patients who achieved an optimal response were categorized as the response group and patients who did not show an optimal response were categorized as the failure group. The failure group of patients included those who progressed to an advanced phase during therapy or those who did not respond to therapy. The morphometry and immunohistochemistry (IHC) profiles were compared between these groups.

Statistical analysis

All categorical variables were summarized as frequency and percentages. Continuous variables were summarized as mean (standard deviation) or median (Q1, Q3) based on normality. The association of various categorical variables between groups was assessed using the chi-square test or Fisher’s exact test. The association of continuous variables that satisfied normality was compared between two groups using the independent T-test and between three groups using a one-way ANOVA. Non-normally distributed data were compared using the Whitney U test between two groups and the Kruskal-Wallis test between three groups. A p-value <0.05 was considered statistical significance. Sensitivity, specificity, positive predictive value (PPV), negative predictive value (NPV), diagnostic accuracy, etc. were summarized in percentages. All statistical analysis was performed by IBM SPSS Statistics for Windows, Version 20 (Released 2011; IBM Corp., Armonk, New York, United States).

## Results

A total of 150 newly diagnosed patients with CML were included in the study. All patients were positive for BCR-ABL1 translocation. The mean (SD) age was 42.9 (13.3) years and 70% were male. At diagnosis, 79% were in the chronic phase, and the rest were in the advanced phase (CML-AdP). All the patients in CML-CP were followed up for a minimum period of 15 months, and 96 out of 119 such patients completed a minimum of 12 months of follow-up.

**Figure 1:**
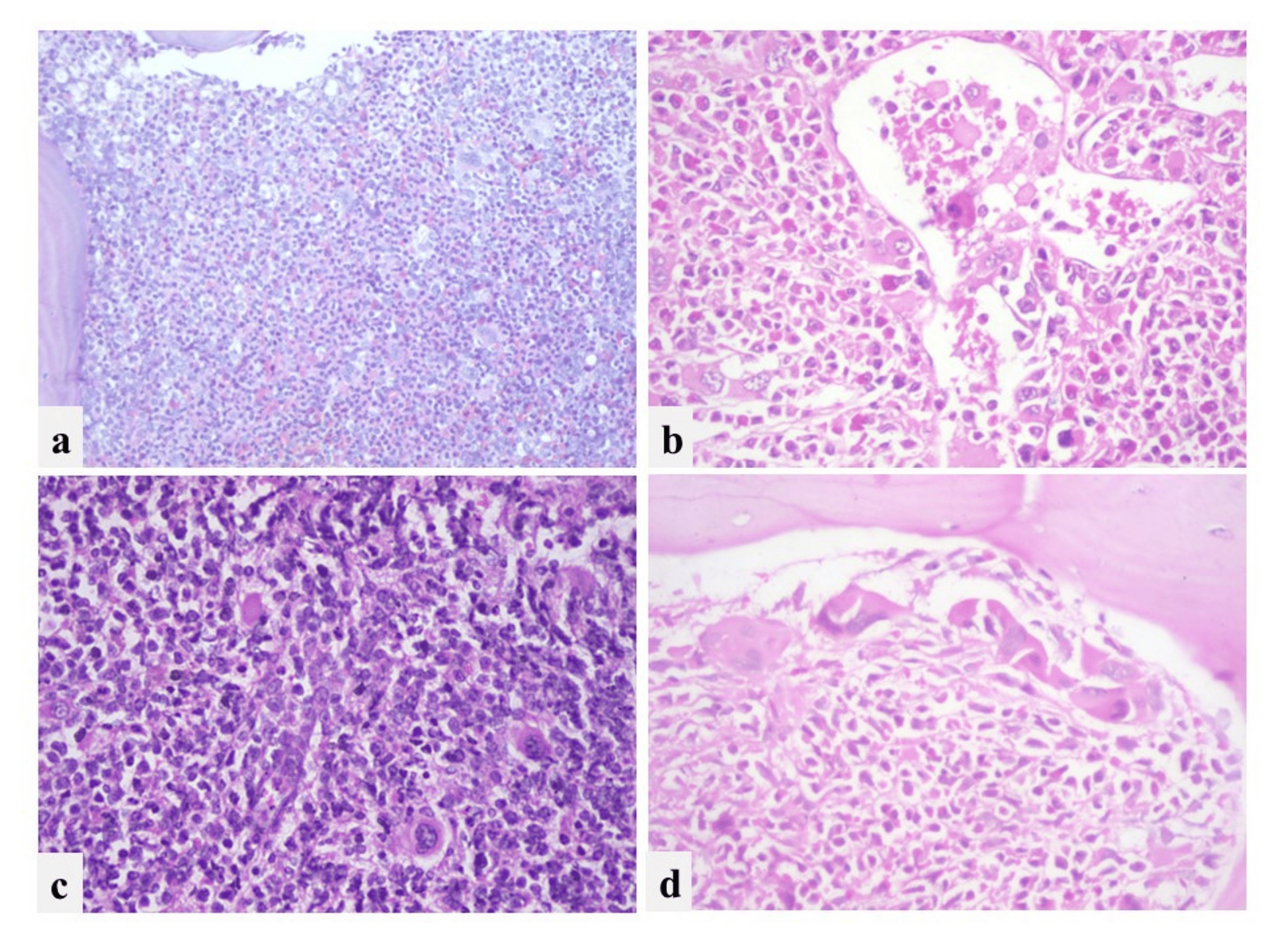
Morphological features of megakaryocytes: a) Dwarf megakaryocytes distributed in the interstitium (40x), b) Megakaryocytes in sinusoid (400x), c) Scattered distribution of megakaryocytes (250x), d) Paratrabecular clustering (400x)

Splenomegaly was seen in 92% of cases with massive splenomegaly in 53% of the patients. The morphology of megakaryocytes was analyzed on H&E-stained sections for all the cases. Megakaryocytic hyperplasia was noted in 78% of the cases. The distribution pattern of megakaryocytes was scattered in 67% of the cases, clusters of megakaryocytes were seen in 21% of cases, and in 12% of cases, both scattered and clusters were seen (Figure 1).

Dwarf megakaryocytes were seen in 98% of the cases, and enlarged and pleomorphic forms in 24%. Monolobed megakaryocytes were seen in 15% of cases and 3% of cases showed megakaryoblasts. The location of megakaryocytes was in the interstitium in 99% of cases, paratrabecular in 37% of cases, and in 14% of cases, it was seen in sinusoids.

**Figure 2:**
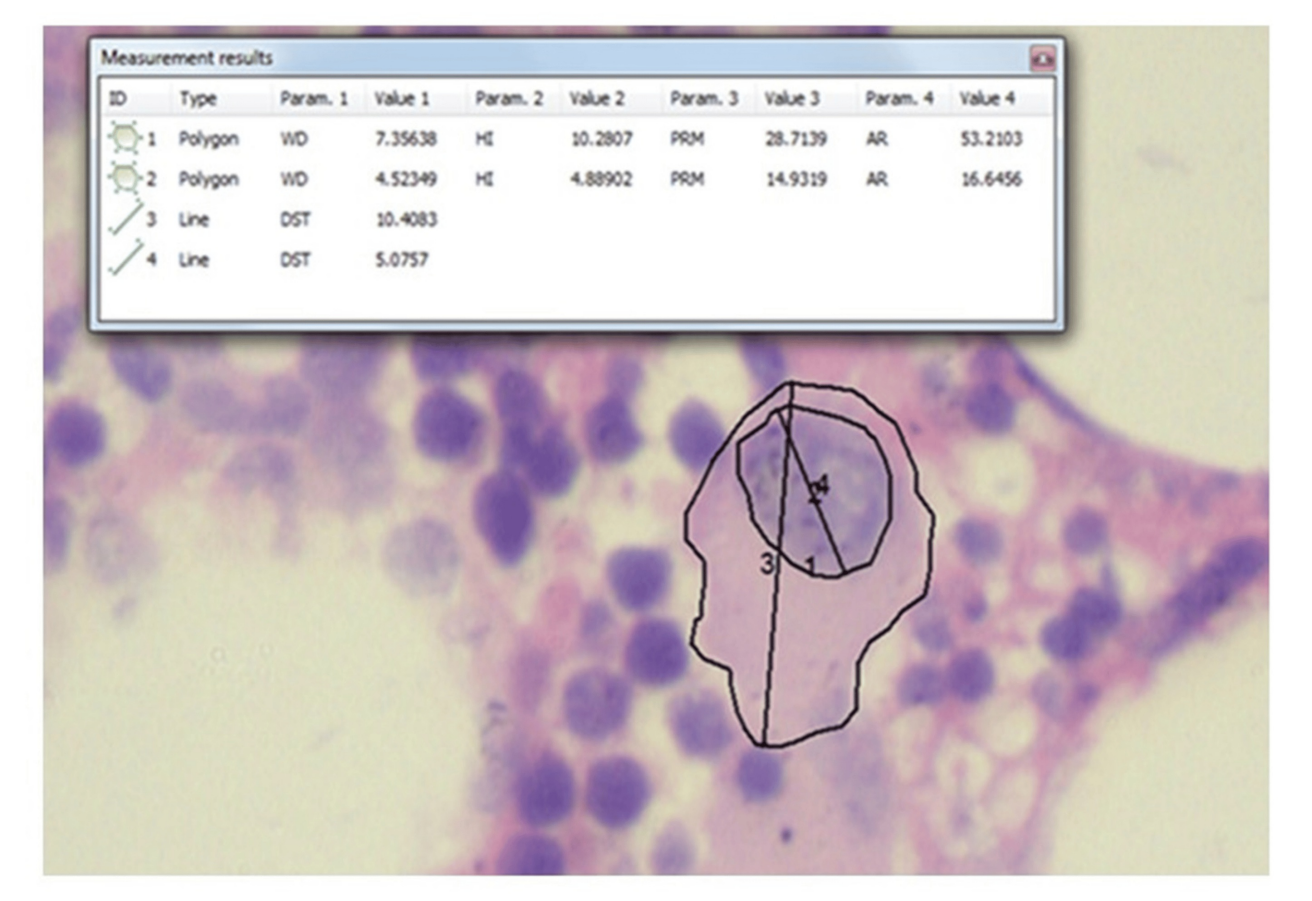
Photomicrograph showing megakaryocyte morphometric assessment of megakaryocytes on H&E. 1) Cytoplasmic area; 2) Nuclear area; 3) Cytoplasmic major diameter; 4) Nuclear major diameter (400x)

The megakaryocyte morphometry and megakaryocyte count were done on 128 cases. The median megakaryocyte count was 2947 per mm^2^ (Q1, Q3: 2189-4695). The mean (SD) of the cytoplasmic area was 201.6 (62.28) µm^2^ and the mean (SD) of the nuclear area was 58.4 (20.98) µm^2^. The mean (SD) of cytoplasmic and nuclear major diameters were 20.03 (5.21) µm and 10.98 (2.98) µm, respectively. The mean (SD) N:C ratio of megakaryocytes in CML was 0.29 (0.062). The results are summarized in Table 1 and a representative of morphometry is given in Figure 2.

**Table 1:** Megakaryocyte morphometry

Parameters	n	Mean	SD
Cytoplasmic area (µm^2^)	128	201.6	62.28
Nuclear area (µm^2^)	128	58.4	20.98
N:C ratio	128	0.29	0.062
Parameters	n	Median	Q1,Q2
Cytoplasmic major diameter (µm)	128	19.9	17.6, 21.9
Nuclear major diameter (µm)	128	10.65	9.36, 11.96
Megakaryocytes/mm^2^	128	2947	2189, 4695

Megakaryocyte morphometry parameters and megakaryocyte count were compared between CML-CP and CML-AdP. None of the morphometry parameters showed a significant difference between CML-CP and CML-AdP (Table 2).

**Table 2:** Comparison of megakaryocytic morphometry in phases of CML Statistical significance: p-value < 0.05 CML: chronic myeloid leukemia; CML-CP: CML in the chronic phase; CML-AdP: CML in the advanced phase

Variables	CML-CP (n = 103)	CML-AdP (n = 25)	Statistical significance (p-value)
Cytoplasmic area (µm^2^), mean (SD)	204.87 (57.8)	187.85 (99.96)	0.313
Nuclear area (µm^2^), mean (SD)	58.76 (19.86)	56.9 (25.51)	0.052
N:C ratio, mean (SD)	0.29 (0.061)	0.305 (0.064)	0.584
Cytoplasmic major diameter (µm), median (Q1, Q3)	19.96 (17.88, 21.83)	19.95 (14.69, 22.16)	0.352
Nuclear major diameter (µm), median (Q1, Q3)	10.82 (9.38, 11.92)	10.40 (8.69, 13.09)	0.548
Megakaryocytes/mm^2^, median (Q1, Q3)	2863 (2105, 4168)	3874 (2526, 5053)	0.073

Immunohistochemistry on megakaryocytes at diagnosis

**Figure 3:**
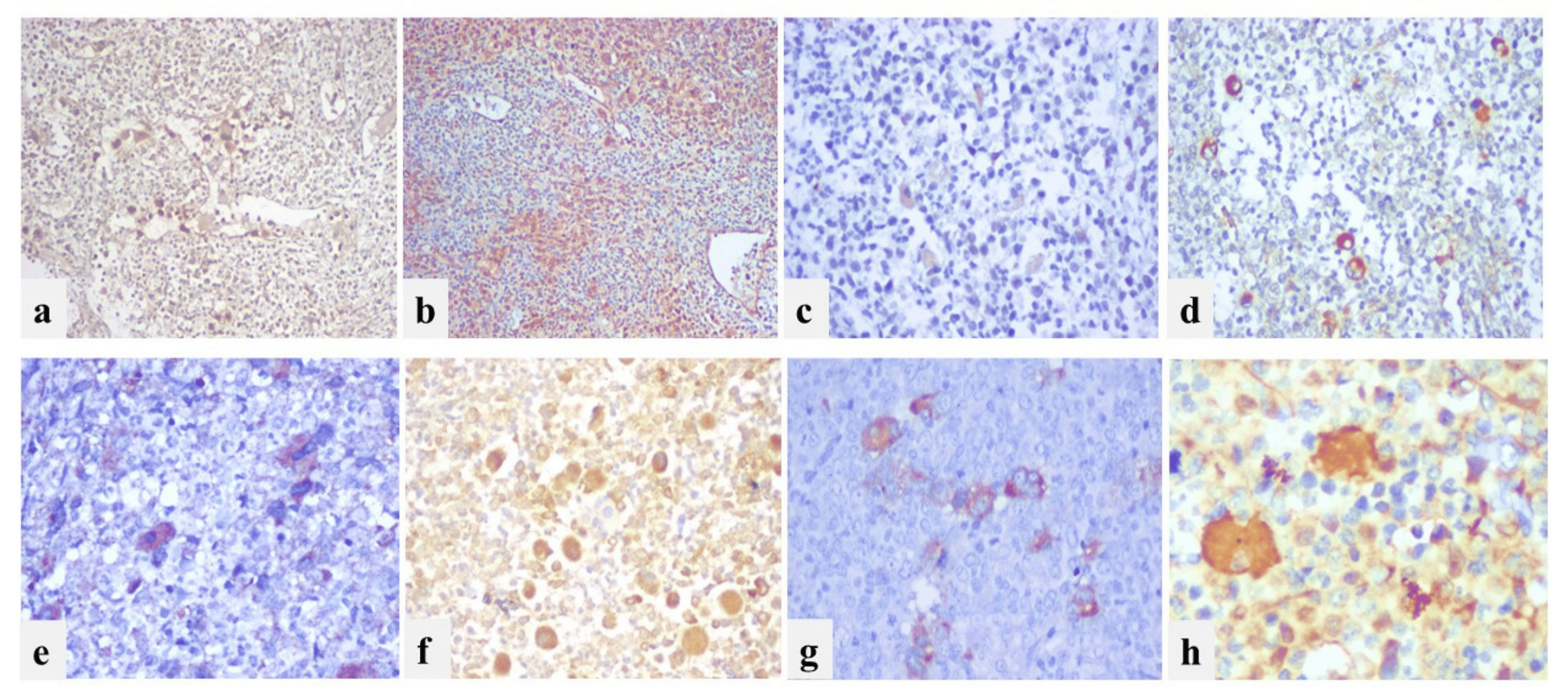
IHC profile of megakaryocytes: megakaryocytes positive for a) VEGF (40x), b) FOXP3 (40x), c) N-cadherin (100x), d) CXCL12 (100x), e) CD48 (250x), f) CD44 (250x), g) PDL-1 (250x), h) IL-7 (400x) IHC: immunohistochemistry; VEGF: vascular endothelial growth factor

In our study, VEGF, FOXP3, osteopontin, N-cadherin, CXCL12, CD150, CD48, CD44, PDL-1, and IL-7 were expressed on megakaryocytes but a chi-square test of independence did not show any significant association with CML-CP and CML-AdP. The expression of most of the markers was strong and cytoplasmic. The results are summarized in Table 3 and the representative images for marker expression are given in Figure 3.

**Table 3:** Expression pattern of IHC markers on megakaryocytes Statistical significance: p-value < 0.05 IHC: immunohistochemistry

Expression of markers on megakaryocytes		CML-CP (n = 119)	CML-AdP (n = 31)	Statistical significance (p-value)
VEGF	Positive	53 (44.5)	15 (48.4)	0.84
	Negative	66 (55.5)	16 (51.6)	
FOXP3	Positive	23 (19.3)	5 (16.1)	0.80
	Negative	96 (80.7)	26 (83.9)	
Osteopontin	Positive	22 (18.5)	8 (25.8)	0.45
	Negative	97 (81.5)	23 (74.2)	
N-cadherin	Positive	38 (31.9)	12 (38.7)	0.52
	Negative	81 (68.1)	19 (61.3)	
CXCL12	Positive	95 (79.8)	26 (83.9)	0.79
	Negative	24 (20.2)	5 (16.1)	
CD150	Positive	13 (10.9)	4 (12.9)	0.75
	Negative	106 (89.1)	27 (87.1)	
CD48	Positive	49 (41.2)	12 (38.7)	0.84
	Negative	70 (58.8)	19 (61.3)	
CD44	Positive	5 (4.2)	2 (6.5)	0.63
	Negative	114 (95.8)	29 (93.5)	
PDL1	Positive	43 (36.1)	14 (45.2)	0.41
	Negative	76 (63.9)	17 (54.8)	
IL7	Positive	30 (25.2)	6 (19.4)	0.64
	Negative	89 (74.8)	25 (80.6)	

Megakaryocyte morphometry in treatment outcomes

Of the 95 patients on follow-up, morphometric data was available in 85 patients, of whom 44 responded and 11 and 30 patients progressed or developed resistance, respectively. A Kruskal-Wallis test was performed to evaluate whether the megakaryocyte count, cytoplasmic major diameter, and nuclear major diameter differed between the treatment outcomes. The results indicated that there were no significant differences in megakaryocyte count, cytoplasmic major diameter, or nuclear major diameter between treatment outcomes. A one-way ANOVA test was performed to evaluate whether there was a difference between the megakaryocyte cytoplasmic area, nuclear area, and nuclear-cytoplasmic area of the treatment outcomes, but the results indicated no significant differences in the megakaryocyte cytoplasmic area, nuclear area, or nuclear-cytoplasmic area between treatment outcomes. The results are summarized in Table 4.

**Table 4:** Comparison of megakaryocyte morphometric parameters between treatment outcomes Statistical significance: p-value < 0.05

Morphometric parameters	Progression (n = 11)	Resistance (n = 30)	Response (n = 44)	Statistical significance (p-value)
Megakaryocyte count/mm^2^, median (Q1, Q3)	2400 (1768, 3115)	2863 (1852, 4147)	3431 (2210, 5305)	0.066
Cytoplasmic major diameter (µm), median (Q1, Q3)	20.6 (17.7, 21.9)	20.4 (18.6, 22.5)	19.6 (17.4, 21.0)	0.162
Nuclear major diameter (µm), median (Q1, Q3)	11.5 (9.9, 12.4)	10.6 (9.4, 11.6)	10.6 (9.3, 11.8)	0.298
Cytoplasmic area (µm^2^), mean (SD)	224.5 (57.7)	214.1 (39.1)	189.8 (57.3)	0.052
Nuclear area (µm^2^), mean (SD)	66.9 (19.1)	59.04 (15.9)	56.2 (20.6)	0.249
Nuclear-cytoplasmic ratio (NC ratio), mean (SD)	0.302 (0.056)	0.274 (0.044)	0.299 (0.065)	0.153

Association of expression of markers on megakaryocytes with treatment outcome

A chi-square test of independence found no significant relationship between VEGF expression in megakaryocytes and treatment outcomes. Fisher’s exact test showed no significant relationship between FOXP3-positive megakaryocytes and treatment outcomes (p = 0.15). Similarly, no statistical relationship was found between OPN-expressing megakaryocytes and treatment outcomes (p = 0.191) or between N-cadherin-expressing megakaryocytes and treatment outcomes (p = 0.48). The chi-square test showed no association for CD150 and CD48 expressions with treatment outcomes (p = 0.062 and p = 0.583, respectively).

However, Fisher’s exact test revealed a significant relationship between CD44-positive megakaryocytes and treatment outcomes (p = 0.001). Notably, 80% of CML-CP cases with CD44-positive megakaryocytes progressed to an advanced phase, while none showed therapy resistance. Fisher’s exact test also found no statistical association between CXCL12, PDL1, and IL7 expression on megakaryocytes and treatment outcomes. The results are summarized in Table 5.

**Table 5:** Association of megakaryocyte expression and treatment outcomes Statistical significance: p-value < 0.05; *: statistical significance

Expression of markers on megakaryocytes		Progression (n = 12)	Resistance (n = 33)	Response (n = 51)	Statistical significance (p-value)
VEGF	Negative	6 (50)	19 (57.6)	29 (56.9)	0.89
	Positive	6 (50)	14 (42.4)	22 (43.1)	
FOXP3	Negative	7 (58.3)	26 (78.8)	43 (84.3)	0.15
	Positive	5 (41.7)	7 (21.2)	8 (15.7)	
Osteopontin	Negative	7 (58.3)	28 (84.8)	41 (80.4)	0.19
	Positive	5 (41.7)	5 (15.2)	10 (19.6)	
N-cadherin	Negative	9 (75)	26 (78.8)	34 (66.7)	0.48
	Positive	3 (25)	7 (21.2)	17 (33.3)	
CXCL12	Negative	2 (16.7)	6 (18.2)	10 (19.6)	1.00
	Positive	10 (83.3)	27 (81.8)	41 (80.4)	
CD150	Negative	11 (91.7)	32 (97)	41 (80.4)	0.062
	Positive	1 (8.3)	1 (3)	10 (19.6)	
CD48	Negative	8 (66.7)	19 (57.6)	26 (51)	0.58
	Positive	4 (33.3)	14 (42.4)	25 (49)	
CD44	Negative	8 (66.7)	33 (100)	50 (98)	0.001^*^
	Positive	4 (33.3)	0 (0)	1 (2)	
PDL1	Negative	9 (75)	20 (60.6)	32 (62.7)	0.75
	Positive	3 (25)	13 (39.4)	19 (37.3)	
IL7	Negative	9 (75)	25 (75.8)	36 (70.6)	0.94
	Positive	3 (25)	8 (24.2)	15 (29.4)	

Accuracy of CD44 positive megakaryocytes predicting the progression

The CD44-expressing megakaryocytes were significantly associated with the treatment outcome, especially with progression. Hence, the predictive accuracy of CD44-expressing megakaryocytes to predict the progression of disease was calculated. The sensitivity, specificity, PPV, and NPV were calculated for CD44-positive megakaryocytes in predicting progression. The sensitivity and specificity for CD44-expressing megakaryocytes in predicting progression were 33.3% and 98% with a PPV of 80% and NPV of 96.1% (Table 6).

**Table 6:** Diagnostic accuracy of CD44 positive megakaryocytes predicting the progression PPV: positive predictive value; NPV: negative predictive value

Variable/category		Outcome		Sensitivity (%)	Specificity (%)	PPV (%)	NPV (%)
Variable	Category	Progression	Response				
CD44 on megakaryocytes	Positive	4	1	33.3	98	80	96.1
	Negative	8	50				

## Discussion

CML is a clonal disorder of HSCs, commonly manifested in the chronic phase of the disease. Patients eventually progress to the advanced phase of the disease, including the blast crisis [14,15].

The hyperplasia of megakaryocytes in 78% of the cases is a finding commonly associated with MPN. The predominance of megakaryocytes is consistent with the bone marrow remodeling observed in these diseases [16]. The observation of dwarf megakaryocytes in 98% of cases was consistent with previous studies [17-19]. In our study, 37% of megakaryocytes were paratrabecular, and 14% of cases had an intra-sinusoidal distribution. When megakaryocytic clusters were evaluated, 21% of cases showed clusters, 67% had scattered megakaryocytes, and 12% had both clustered and scattered megakaryocytes. When compared with a previous study, the clustered megakaryocytes are in concordance [17].

The median megakaryocyte count was determined to be 2947 per mm^2^ (Q1, Q3: 2189, 4695), indicating a wide variability across the cases [17]. The morphometric analysis revealed a mean cytoplasmic area of 201.6 µm^2^ with an SD of 62.28 µm^2^ and a mean nuclear area of 58.4 µm^2^ with an SD of 20.98 µm^2^. The mean cytoplasmic major diameter was 20.03 µm (SD: 5.21 µm), while the mean nuclear major diameter was 10.98 µm (SD: 2.98 µm). A similar result in CML was reported in a previous study [20]. The calculation of the N:C ratio was particularly interesting, which showed a mean value of 0.29 (SD: 0.062) for megakaryocytes in CML. This ratio reflects the relative sizes of the nucleus and cytoplasm in megakaryocytes, providing insights into their maturation and functional status. The findings suggest significant heterogeneity in megakaryocyte parameters within CML cases, possibly reflecting the disease's diverse biological behavior and clinical presentations. The morphometry parameters did not show any difference between the chronic and advanced phases of CML.

Our study found no association between megakaryocyte morphometry parameters and treatment outcomes. The megakaryocyte count was reduced in the progression and resistance groups compared to the response group. Similarly, the cytoplasmic area of megakaryocytes was greater in progression and resistance compared to the response group, but the differences were not statistically significant.

The megakaryocytes showed VEGF, FOXP3, osteopontin, N-cadherin, CXCL12, CD150, CD48, CD44, and PDL1, as well as IL7 positivity in different phases of CML cases. The staining reaction was not statistically associated with the phases of CML.

Only CD44-stained megakaryocytes showed a significant association with treatment outcomes; none of the other marker-positive megakaryocytes showed any statistical association with treatment outcomes.

CD44 has many roles in the BMM in CML, including intracellular signal transmission for cell proliferation, differentiation, and migration. It also contributes to tumorigenesis and acts as a leukemic stem cell [21]. CD44 acts as a target for β-catenin, osteopontin, and hyaluronic acid [21]. Grosso et al. reported an upregulation of CD44 in treatment-resistant CML cells [22].

In the present study, CD44 expression on megakaryocytes showed a significant association with treatment outcomes (p = 0.001), indicating a potential role for CD44 expression on megakaryocytes in predicting treatment response, progression, or resistance. Cases showing CD44-expressing megakaryocytes were higher in the progression group (33.3%) compared to the response (2%) and resistance groups (0%). The predictive accuracy of CD44-positive megakaryocytes for predicting progression was poor, with a low sensitivity of 33.3% and a PPV of 80%.

The limitations of our study include a lack of follow-up bone marrow biopsy to assess changes in IHC markers. Flow cytometry or multiplex immunohistochemistry, which would have helped us identify the expression of the markers on the same cell, could not be done in our study.

## Conclusions

Megakaryocyte morphology and morphometry were heterogeneous in CML; however, these parameters did not show any significant association with phases of CML as well as the treatment outcomes. Detailed immunohistochemical staining of the various markers of the microenvironment was done. It helped highlight the presence or absence of these markers on megakaryocytes during different phases and treatment outcomes in CML. None of them showed any association with the phases of CML. Apart from CD44, none of the markers showed a significant association with treatment outcome as well. The CD44 expression on megakaryocytes showed a significant association with treatment outcome; patients who showed progression had CD44-positive megakaryocytes, possibly reflecting its role in resistance and progression of the disease.
